# Participant recruitment and retention in a longitudinal study: experience from SARS-CoV-2 cohort in Ethiopia

**DOI:** 10.1186/s12874-026-02823-2

**Published:** 2026-03-14

**Authors:** Eyob Girma Abera, Yeneneh Berhanu Kebede, Zeleke Alemu Adulo, Neway Tadesse Tilahun, Bikila Alemu Kebede, Zerihun Asefa Hordofa, Wondimagegn Adissu Maleko, Daniel Yilma, Esayas Kebede Gudina

**Affiliations:** 1 Department of Public Health, Jimma University, Jimma, Oromia Ethiopia; 2 Clinical Trial Unit, Jimma University, Jimma, Oromia Ethiopia; 3 Jimma Medical Center, Jimma University, Jimma, Oromia Ethiopia; 4 School of Medical Laboratory Sciences, Jimma University, Jimma, Oromia Ethiopia; 5 Department of Internal Medicine, Jimma University, Jimma, Oromia Ethiopia

**Keywords:** Longitudinal Study, SARS-CoV-2 Antibody Responses, Participant Retention, Ethiopia, Cohort Study, Recruitment Strategies

## Abstract

**Background:**

Longitudinal cohort studies are essential for understanding changes in health over time, yet participant recruitment and retention remain major challenges, especially in low-resource settings. This study describes the strategies, outcomes, and lessons learned from a two-year longitudinal severe acute respiratory syndrome coronavirus 2 (SARS-CoV-2) cohort study conducted in Ethiopia, focusing on engagement among healthcare workers (HCWs) and community members.

**Methods:**

This prospective observational cohort study was nested within a larger longitudinal SARS-CoV-2 project conducted from November 2022 to December 2024. The study enrolled 500 participants, including 350 HCWs and 150 community members, at Jimma Medical Center (JMC). Participants were followed every three months over two years to assess SARS-CoV-2-specific antibody responses. Data collection included venous blood samples, nasopharyngeal swabs (NPS) during symptomatic episodes, and clinical and demographic information. Recruitment used multiple approaches such as phone calls, in-person outreach, and community visits. Retention strategies included flexible scheduling, regular communication, voluntary home visits, and engagement by culturally aware, participant-focused staff.

**Results:**

The study reached 100% of its target enrollment within 48 days. Over the two-year follow-up, the overall retention rate was 88%, with 82% of participants completing all follow-up visits. High retention was supported by structured contact tracking, regular reminders, culturally sensitive communication, and fair participant compensation. A total of 60 participants were lost to follow-up. Among healthcare workers, the main reasons were relocation (36, 65.5%), health-related issues (6, 10.9%), and unstated reasons (7, 12.7%), with smaller numbers lost due to religious considerations, family influence, or death. In the community group, attrition was mainly due to health-related issues (2, 40%), relocation (1, 20%), religious perspectives (1, 20%), and family influence (1, 20%).

**Conclusion:**

High retention is achievable in longitudinal studies in resource-limited settings when supported by well-planned, context-specific recruitment and retention strategies. This study highlights the value of transparency, participant-centered practices, and operational flexibility, offering practical guidance for future public health research in low- and middle-income countries.

**Supplementary Information:**

The online version contains supplementary material available at 10.1186/s12874-026-02823-2.

## Introduction

Longitudinal cohort studies facilitate the tracking of changes within individuals over time, providing critical insights into dynamic biological, clinical, behavioral, and social processes [1]. By following individuals through repeated observations, these studies enable the investigation of temporal patterns, causal relationships, and long-term outcomes that cannot be captured in cross-sectional designs [2]. It provides a powerful framework to explore how exposures, interventions, and social determinants shape patterns of health and illness over time [3].

Effective recruitment is a crucial first step for successful longitudinal studies. Evidence from community-based and resource-limited settings demonstrates that multipronged outreach strategies, culturally tailored communication, and close engagement with stakeholders improve enrollment rates and participant diversity [4, 5]. Reviews of cohort studies also highlight that participant-friendly recruitment approaches, including clear consent procedures and flexibility in scheduling, enhance both recruitment and subsequent retention [6].

Despite their strengths, longitudinal studies are inherently complex and resource-intensive, requiring sustained engagement with participants and robust systems for data collection [7]. While these challenges exist globally, they are often more effectively managed in high-income countries, where research infrastructure, digital systems, and reliable funding streams help support participant follow-up. In contrast, studies in low- and middle-income countries (LMICs) face major challenges due to logistical, infrastructural, and contextual barriers that can affect study implementation [8]. A particularly critical aspect of this complexity lies in participant recruitment and retention. Longitudinal studies frequently encounter difficulties in maintaining consistent participation, with systematic attrition more common among older individuals, have limited education, or face multiple health issues [9, 10]. The extended duration and repeated assessments inherent in these studies can place a considerable burden on participants, contributing to attrition over time [6]. One key issue is research fatigue, which occurs when participants feel overwhelmed by the demands of ongoing involvement in the study which can lead to decreased motivation and engagement over time, ultimately affecting the quality and reliability of the research outcomes [11].

Despite these obstacles, effective retention strategies are essential to ensure the validity and reliability of study outcomes [6]. Established retention strategies within longitudinal cohort studies include: cash or gift incentives, sending reminders to participants, and offering alternative methods of data collection [5]. Maximizing cohort retention in longitudinal research not only reduces administrative costs but also enhances the efficiency of research processes. Ensuring continued participant engagement throughout the study helps minimize potential outcome biases and enhances the validity of the findings. Adopting an evidence-based cohort retention framework is crucial, as it provides a structured approach to addressing barriers to retention. This framework helps researchers maintain consistent participant engagement, leading to more reliable and generalizable results.

The parent study was a two-year longitudinal cohort established at Jimma Medical Center (JMC) to monitor severe acute respiratory syndrome coronavirus 2 (SARS-CoV-2) infection, reinfection, and antibody dynamics among healthcare workers (HCWs) and community members. The study was initiated during a period of ongoing viral circulation, limited vaccine coverage, and the emergence of new variants, making long-term immune surveillance an important priority in the Ethiopian context. Generating reliable evidence from such cohorts depends heavily on sustained participant engagement, which places recruitment and retention at the center of successful implementation.

This manuscript focuses on the recruitment and retention experience within the broader SARS-CoV-2 cohort. We describe the approaches used to enroll and retain participants and reflect on the key challenges and lessons that shaped the study’s completion.

## Methods

### Study design

This was a prospective, observational cohort nested within a larger longitudinal SARS-CoV-2 project conducted from November 2022 to December 2024. The present analysis focused on evaluating participant recruitment and retention within this cohort.

### Operational definitions

- Missed Visit: A visit is considered missed if a participant fails to attend the scheduled appointment and does not present within six weeks of the appointment date. However, the participant remains eligible if they attend the subsequent scheduled visit. Only one missed visit is permitted for continued participation in the study.
- Discontinuation: Voluntary withdrawal from the study by the participant at any time, for any reason, including but not limited to personal choice, lack of interest, or refusal to provide a reason for withdrawal.
- Forced withdrawal: Mandatory removal from the study due to missing two or more scheduled visits, regardless of the participant’s willingness to continue, in accordance with the study protocol.

### The SARS-CoV-2 study

The main objective of the cohort study was to evaluate the prevalence and incidence of SARS-CoV-2 infection and reinfection, antibody dynamics, and variant-specific neutralization assays, as well as to determine the burden and patterns of other respiratory tract infections among HCWs and community members in Ethiopia.

The study involved the recruitment of 350 HCWs working at JMC and 150 community members residing in Jimma Town. Participants were aged 18 years or older and willing to provide blood samples via venipuncture for serological and immunological analyses. Individuals willing to report flu-like symptoms during the study period were also included. Prisoners and individuals who were critically ill at enrollment were excluded.

Upon enrollment, participants were scheduled for follow-up visits every three months, resulting in a total of nine rounds over two years, from November 2022 to December 2024. These visits included collection of 15–19 mL of blood for antibody testing. Nasopharyngeal swabs (NPS) were collected for viral testing when participants reported flu-like symptoms, and unscheduled visits were arranged for participants presenting with symptoms between scheduled appointments (Additional file 1).

### Study site

This study was implemented at the Jimma University Clinical Trial Unit (JUCTU), located in the main campus of Jimma University in Jimma, Ethiopia, approximately 350 km southwest of the capital, Addis Ababa. JUCTU was established in 2013 and evolved into a well-established clinical research unit. It is staffed by 15 research and administrative personnel, including two senior researchers, two mid-level researchers, a study coordinator, four Doctor of philosophy (PhD) students, a study nurse, laboratory professionals, and other support staff. The unit has conducted multiple research projects in collaboration with both local and international partners including phase II and III drug development and diagnostic validation studies.

### Recruitment and selection of HCWs

Recruitment of HCWs at JMC followed a multipronged approach designed to reach all staff and avoid information gaps. We began by contacting individuals who had participated in the previous SARS-CoV-2 seroepidemiology study (2020–2022) [12, 13]. They were called, briefed about the new cohort, and invited for an in-person discussion if they were interested.

At the same time, the outreach coordinator and study nurse visited clinical units to engage directly with unit heads. Each head received a short study summary and was asked to compile a list of HCWs willing to participate, including their profession, department, and phone number (Additional file 2). Staff were clearly informed that registering interest did not guarantee enrollment and that eligibility would be confirmed during screening.

For senior clinicians who were harder to reach during ward visits, email invitations were sent with a brief explanation of the study and a contact phone number to facilitate follow-up.

Selection of HCWs began once the recruitment activities produced a pool of interested participants. Priority was first given to individuals who had taken part in the 2020–2022 seroepidemiology cohort and indicated interest in continuing, as long as they met eligibility criteria. All willing volunteers from this group were included. To complete the required sample size, additional HCWs were drawn from those recruited through unit-level outreach and email communication. When the number of new volunteers exceeded the remaining slots, a structured sampling frame based on professional category and departmental distribution was created to ensure balanced representation. A lottery method was then used to select from this group. If a selected individual declined or could not be reached, the next person on the registration list was approached.

### Recruitment and selection of community members

Community members were recruited using phone outreach and home visits, targeting both individuals previously enrolled in the 2020–2022 cohort [12, 13] and new volunteers from different areas of Jimma Town. Initial phone calls were made to former participants; although many contacts were inactive, a small number agreed to discuss the study further, while others declined participation.

To reach a broader and more diverse pool, the outreach team selected villages using a purposive and stratified approach to represent different directions within the town. Within these villages, households were approached, and individuals who expressed interest were registered. The coordinator and study nurse provided detailed study information and documented key details, including name, phone number, residence, and a designated local contact to assist with future follow-up (Additional file 3). Assigning a local contact facilitated communication with participants who might relocate or become temporarily unreachable.

Selection of community participants followed a village-based approach, whereby sampling was conducted separately within each selected village to ensure geographic representation across Jimma Town. In villages where only a small number of individuals registered (about 10–15 people), all eligible participants were included. In villages with larger numbers of registrants, a lottery method was used to select participants proportional to the required sample size. Backup lists were prepared to replace individuals who declined or could not be reached during enrollment.

### Recruitment of participants living with HIV

The study included a cohort of participants living with HIV from both HCWs and community groups. The outreach coordinator, study nurse, and study coordinator collaborated with staff at the Antiretroviral Therapy (ART) clinic at JMC. Clinic staff were informed about the research objectives and tasked with identifying and registering willing participants from both HCWs and community members. The registration form included participants’ names, phone numbers, village of residence, and an alternate contact person in case of communication challenges.

### Informed consent and enrollment

Prior to enrollment, a thorough and participant-friendly consenting process was conducted. Participants were provided with detailed information about the study procedures in an understandable manner, ensuring they were fully aware of all requirements, including blood sample collection across nine follow-up visits. To enhance comprehension and reassure participants about the safety of blood collection, a visual demonstration was used: jars containing colored liquid illustrated the total volume of blood in the human body (5 L), the volume drawn during routine blood donation (450 mL), the total volume to be collected over the study period (147 mL), and the small volume collected at each visit (15–19 mL). This approach addressed common concerns regarding blood volume. Participants were given clear explanations, opportunities to ask questions, and reassurances that they could decline participation or withdraw from the study at any time without consequences.

### Participant retention strategies

To ensure participant retention, the following strategies were implemented:

1. Contact locator system and tracing mechanism: These were implemented to trace participants who might have been lost to follow-up. This process involved registering participants’ phone numbers along with detailed information about their specific home locations. Additionally, participants were requested to provide contact details for two alternative close contacts who could be reached if their primary phone number became inactive.
  In cases where participants could not be reached, a three-step tracing approach was implemented before classifying them as lost to follow-up:
  Step 1: The contact locator system was consulted to reach the two alternate individuals provided by the participant.
  Step 2: For HCWs, their respective unit heads were contacted, while a designated staff member facilitated tracing for community members.
  Step 3: If the first two steps were unsuccessful, a visit to the participant’s registered home location was made as the final effort before concluding their status.
2. Team meeting: The project team held meetings every two weeks to assess study progress, address emerging challenges, and plan upcoming activities. A key focus of these meetings was participant retention, with discussions centered on strategies to ensure consistent follow-up across all scheduled visits. The team regularly evaluated engagement approaches and provided direction on how to strengthen communication and maintain active participation throughout the study.
3. Participant communication and engagement strategies: Transparent and consistent communication was central to the study’s retention strategy. From the beginning, participants were clearly informed that the study was observational and not a clinical trial, and that no medications would be provided. Participation was entirely voluntary, and the importance of their role in advancing the understanding of SARS-CoV-2 antibody responses was emphasized. This early transparency helped manage expectations and foster trust between participants and the study team.
  Participants received their test results through two different approaches. For those who provided NPS, results were communicated promptly via phone calls as soon as laboratory processing was complete. In contrast, antibody test results were shared upon participants’ inquiries or during their subsequent study visits.
  The outreach coordinator (EGA) and the study nurse (YBK) were responsible for all participant communications throughout the study. They maintained regular, personalized contact, beginning with a reminder one week before each scheduled visit and followed by another reminder the day before. Communication was primarily conducted through phone calls and text messages using the contact numbers collected during initial registration. All messages and calls were made from the study team’s designated phone number, which was also printed on invitation flyers (Additional file 4). This approach helped participants recognize the caller and enabled direct contact with the study team when needed. Consistent use of a single contact number fostered trust, reduced confusion, and ensured smooth, two-way communication.
  Each contact served multiple functions: confirming appointments, sending reminders, informing participants of results, addressing concerns, and reiterating the importance of continued participation. In cases where participants chose to withdraw, the communication remained respectful and cordial.
4. Flexibility during the window period: Recognizing the demanding schedules of HCWs and other participants, flexibility was a key component of the study’s retention strategy. Appointments were scheduled according to participants’ availability, including off-hours and weekends, while adhering to the standard operating procedures, which allowed a six-week window around each scheduled visit. For participants unable to attend within the regular schedule due to work obligations, illness (within the study’s exclusion criteria), personal challenges, or pregnancy, pre-requested volunteer home visits were arranged for sample collection.
5. Consistent and compassionate staff approach: The study team prioritized building a respectful, supportive, and culturally sensitive relationship with participants. Staff engaged with participants empathetically and professionally during each interaction, both at the health facility and during home visits. These home visits were arranged for participants who were unable to attend scheduled visits due to illness, pregnancy, or other challenges. They were not only a means to continue data and sample collection but also strengthened trust and sustained engagement. This approach reflected local cultural values around care and mutual respect, contributing to a positive study experience and high retention.
6. Study team experience, training, simulation: The study team’s prior experience with participant recruitment and retention in similar longitudinal studies played a crucial role in maintaining high retention rates. Lessons learned from these earlier efforts were directly applied to the current study, helping to establish effective communication strategies and participant engagement practices from the outset.
  To ensure smooth implementation, comprehensive two-day training was conducted before the study launch. This training focused on participant-centered communication, ethical considerations, and adherence to study protocols.
  In addition to the initial training, two scenario-based simulations (dry runs) were conducted, one before the baseline visit and another at the end of the first year. These simulations allowed team members, including data collectors and outreach coordinators, to practice real-life scenarios, refine their interaction strategies, and receive feedback. The simulations were instrumental in reinforcing empathetic communication and troubleshooting potential challenges, ultimately strengthening participant engagement across the study period.
7. Compensation for participant time: Individuals were provided with 500 Ethiopian Birr (equal to 10 United States Dollar (USD) in 2022 exchange rate) per visit as reimbursement for their time, effort, and any potential costs incurred, such as transportation. This compensation was designed to ensure fairness and accessibility, enabling participants to continue contributing to the study without undue burden. The amount was carefully determined to reflect local standards and was approved by the Institutional Review Board (IRB) to ensure it aligned with ethical guidelines, avoiding coercion or undue inducement. Participants were fully informed about this reimbursement during the consent process to maintain transparency.

## Results

### Recruitment

Recruitment for the study began on November 3, 2022, and follow-up ended on December 21, 2022. This initial phase focused on enrolling eligible participants into the study cohort before follow-up activities commenced. All recruitment activities were completed within this 48-day period.

**Fig. 1:**
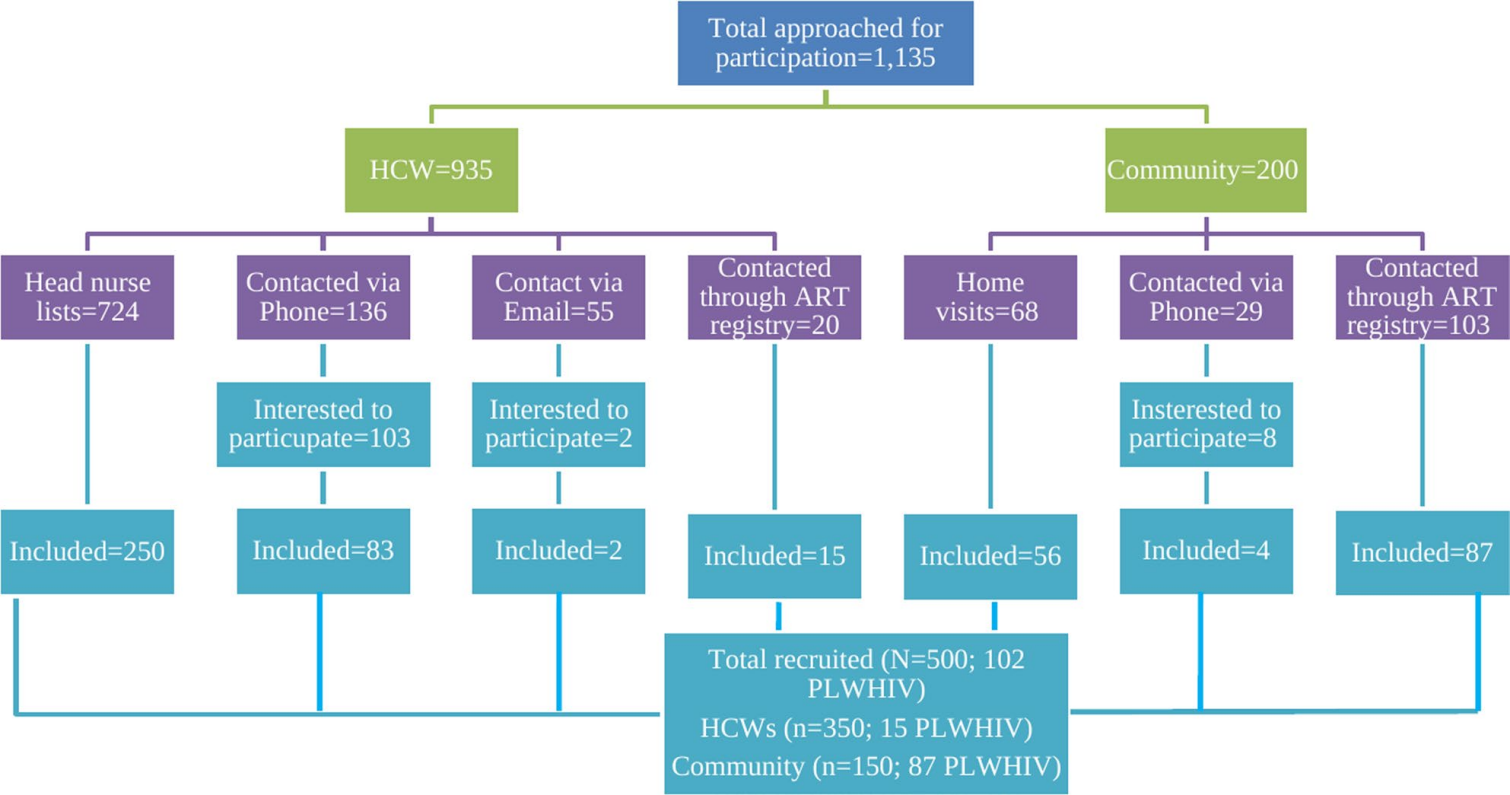
Participants recruitment flow-chart

From the initial pool of individuals contacted through various communication channels, a total of 1,135 participants (935 HCWs and 200 community members) registered and volunteered to take part in the study. Participants living with HIV were intentionally recruited from both groups, with 15 HCWs and 87 community members included. Among the HCWs, the majority of registrations were facilitated by head nurses (*n* = 724), from which 250 participants were ultimately selected, contributing to the targeted sample of 350 HCWs. For the community members, home visits accounted for 68 registrations, leading to the selection of 56 participants. Collectively, these selections yielded the total sample size of 500 participants (Fig. 1).

### Recruited participants baseline characteristics

The median age of participants was 30 years (IQR: 27–37), with females comprising the majority (65.8%). Among the 350 HCWs recruited, nurses or midwives were predominant, accounting for 219 (62.6%). The vast majority of participants (97.6%) had never experienced suspected coronavirus disease 19 (COVID-19), while 2.4% reported a polymerase chain reaction (PCR) confirmed infection. Over half of the participants 269 (53.8%) had received at least one dose of a COVID-19 vaccine. AstraZeneca was the predominant vaccine across all three doses, accounting for 90.7%, 98.3%, and 89.5% of first, second, and third doses, respectively (Table 1).

**Table 1:** Baseline characteristics of participants in the parent SARS-CoV-2 cohort study

Variable	Result (n=500)
Age, *(median, IQR)*	30 (27-37)
Sex, *n (%)*	
Female	329 (65.8)
Male	171 (34.2)
Participant Type, *n (%)*	
Community	150 (30)
Health Care workers	350 (70)
Profession, n (%)*	350 (70)
Nurse/Midwife	219 (62.6)
Laboratory	4 (1.1)
General practitioner	4 (1.1)
Others	123 (35.1)
Any medical condition, *n (%)*	121 (24.2)
HIV status, *Yes*	102 (20.4)
Tuberculosis, *Yes*	36 (7.2)
Hypertension, *Yes*	11 (2.2)
Bronchial Asthma, *Yes*	8 (1.6)
Diabetes Mellitus, *Yes*	7 (1.4)
Ever had suspected COVID-19, *n (%)*	
No	488 (97.6)
Yes, confirmed by PCR	12 (2.4)
COVID-19 vaccination status, *n (%)*	
No	231 (46.2)
Yes, one shot	92 (18.4)
Yes, two shots	120 (24)
Yes, three shots	57 (11.4)
1st Dose, *n (%)*	
AstraZeneca	244 (90.7)
Johnson & Johnson	22 (8.2)
Other	3 (1.1)
2nd Dose, *n (%)*	
AstraZeneca	174 (98.3)
Other	3 (1.7)
3rd Dose, *n (%)*	
AstraZeneca	51 (89.5)
Other	6 (10.5)

*Profession sample (only for HCWs)

*IQR* Interquartile Range, *HIV* Human Immunodeficiency Virus, *COVID-19* Coronavirus Disease 2019, *PCR*Polymerase chain reaction

### Recruitment challenges and mitigation

Challenges were observed during the recruitment phase. Some individuals hesitated to enroll due to limited understanding of the study’s purpose or concerns about participation, even after initially agreeing to the procedures during early registration. In some cases, potential participants needed to consult family members, particularly spouses or elders, before deciding, which led to delays or refusals. To address this, the next person on the registration list was approached to ensure smooth progress in enrollment.

### Retention

**Fig. 2:**
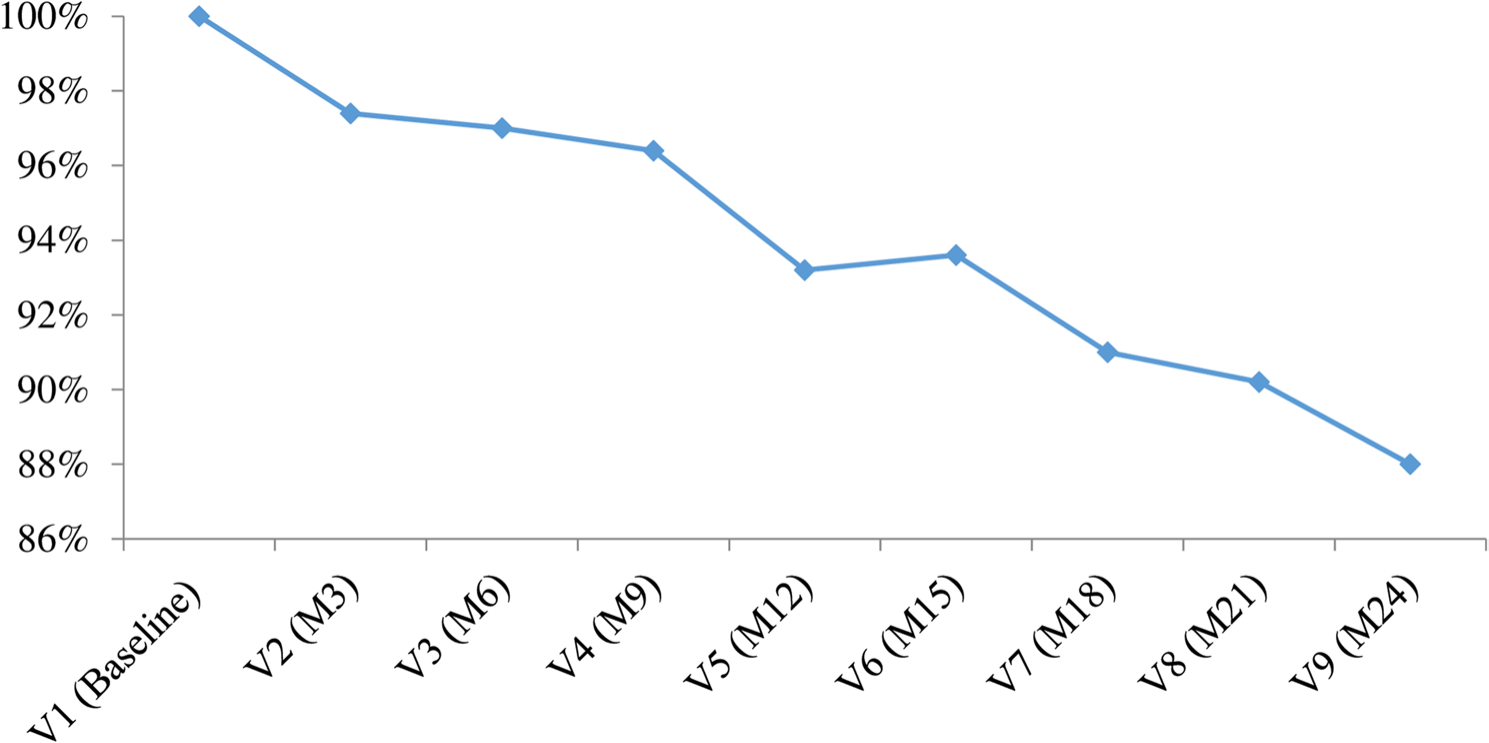
Retention rate across two years of follow-up in a longitudinal cohort study

Starting with an initial cohort of 500 participants, the study achieved 88% (*n* = 440) retention rate at the ninth round of two years follow-up visit. A slight increase in participant retention from 93.2% to 93.6% was observed between the fifth and sixth rounds of follow-up visits. (Fig. 2).

### Participant attrition: baseline profile and reasons

#### Baseline characteristics of participants lost to follow-up

The 60 participants who were lost to follow-up had a median age of 28 years (IQR 26–30). Most were women (65%). The majority were HCWs (*n* = 55, 91.7%), mainly nurses or midwives (68.3%). Among them were two participants living with HIV and one participant each with TB, diabetes and bronchial asthma. Most had never reported suspected COVID-19 (95%). Vaccination coverage in this group was low: 40% were unvaccinated, 30% had received one dose and 25% had received two doses (Table 2).

**Table 2:** Baseline characteristics of participants lost to follow-up (n=60)

Variable	Result (*n* = 60)
Age, *(median*,* IQR)*	28 (26–30)
Sex, n (%)	
Female	39 (65)
Male	21 (35)
Participant Type, *n (%)*	
Community	5 (8.3)
Health Care workers	55 (91.7)
Profession, n (%)*	
Nurse/Midwife	41 (74.5)
Physician	1 (1.8)
Other	13 (23.6)
Level of Education, *n (%)*	
University Degree and Above	47 (78.3)
College	7 (11.7)
High school	4 (6.7)
Primary school	2 (3.3)
Any Medical Conditions, *n (%)*	4 (6.7)
HIV status, Yes	2 (3.3)
Tuberculosis, Yes	1 (1.7)
Bronchial Asthma, Yes	1 (1.7)
Diabetes Mellitus, Yes	1 (1.7)
Ever had suspected COVID-19, *n (%)*	
No	57 (95)
Yes, confirmed by PCR	3 (5)
COVID-19 vaccination status, *n (%)*	
No	24 (40)
Yes, one shot	18 (30)
Yes, three shots	3 (5)
Yes, two shots	15 (25)

*Profession sample (only for HCWs)

*IQR* Interquartile Range, *HIV* Human Immunodeficiency Virus, *COVID-19* Coronavirus Disease 2019, *PCR* Polymerase chain reaction

#### Reasons for participant attrition

**Fig. 3:**
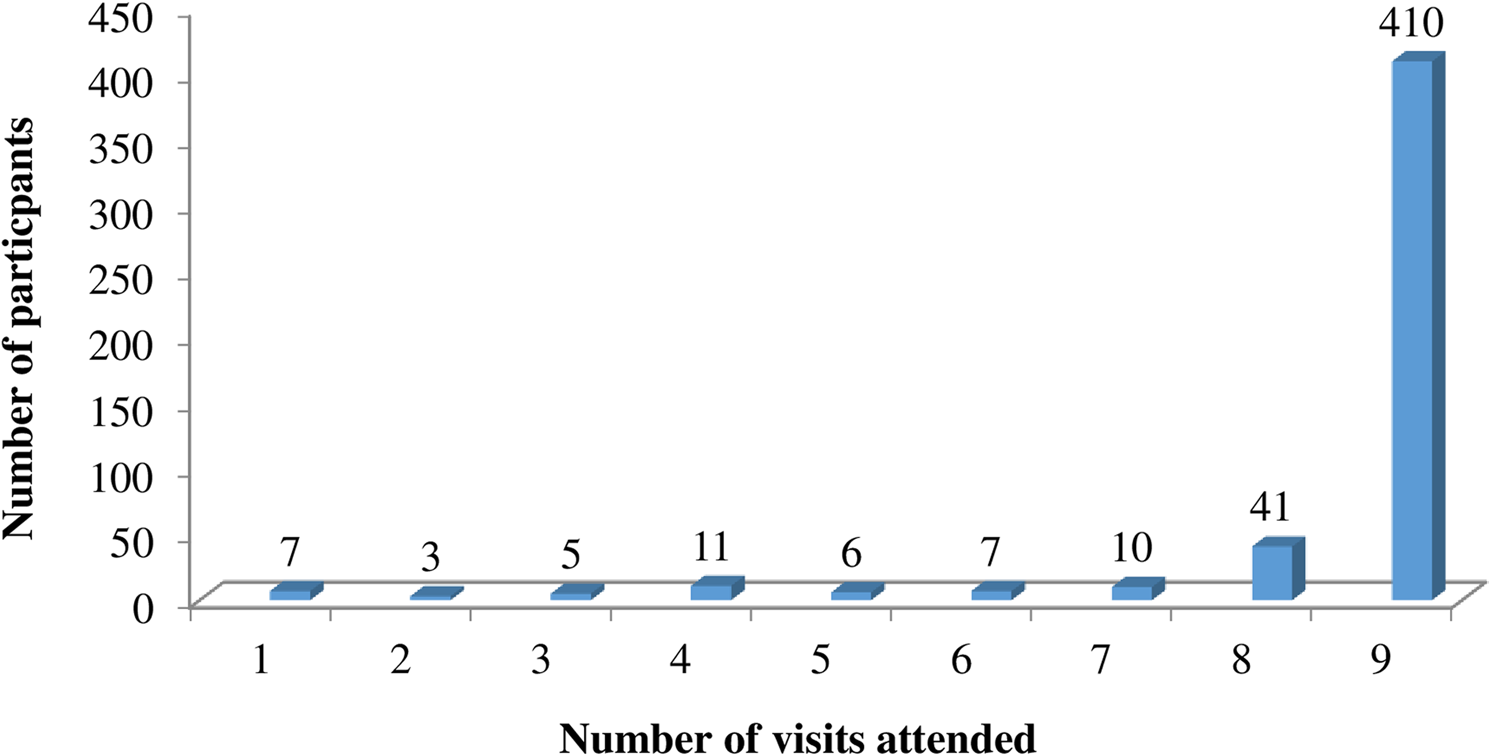
Number of visits attended by participants

Beginning with an initial cohort of 500 participants (350 HCWs and 150 community members), 493 (98.6%) attended at least one follow-up visit beyond baseline, while 7 (1.4%) attended only the baseline round. The majority, 410 (82%), completed all nine scheduled visits (Fig. 3). Retention challenges throughout the study highlighted the complexities of longitudinal cohort research, resulting in the loss of 60 participants (55 HCWs and 5 community members) due to several factors:

1. Relocation: Both local and international relocation posed significant barriers (*n* = 37), preventing participants from attending scheduled visits. The vast majority of those who relocated were HCWs (*n* = 36).
2. Health-related issues: Health complications (*n* = 8) were cited as reasons for discontinuation. These included anemia (*n* = 4; HCWs = 3, community = 1), one community member diagnosed with prostate cancer, and three HCWs who discontinued due to surgery-related conditions.
3. Unstated reasons: Seven HCWs withdrew without providing specific reasons, stating only, “I don’t want to continue the study.”
4. Religious perspectives: Three HCWs and one community member withdrew due to religious objections, with some stating, “Giving blood is forbidden in my religion.”
5. Family influence: Two HCWs and one community member discontinued participation due to family pressure, reporting, “My family are not willing to proceed with the study.”
6. Participant death: One HCW passed away due to an underlying chronic medical condition.

These reasons collectively resulted in the loss of 60 participants over the study period (Table 3).

**Table 3:** Reasons for participant attrition in a longitudinal study on SARSCoV-2 antibody responses

Reasons	HCWs, 55 (91.7%)	Community, 5 (8.3%),
Relocation	36 (65.5)	1 (20)
Health-related issues	6 (10.9)	2 (40)
Unstated reasons	7 (12.7)	0
Religious perspectives	3 (5.5)	1 (20)
Family influence	2 (3.6)	1 (20)
Death	1 (1.8)	0

## Discussion

The study demonstrated that effective participant recruitment, follow-up, and retention strategies can result in high levels of sustained participation, even in the face of challenges commonly encountered in longitudinal cohort studies. This was evident in our assessment of the SARS-CoV-2 antibody response study, which achieved complete recruitment within 48 days with 88% retention rate across nine rounds of follow-up visits over a two-year period among HCWs and community members in Ethiopia.

This study successfully recruited 500 participants (HCWs and community members) within 48 days using multi-modal communication strategies (phone calls, emails, facility and home-based outreach), aligning with longitudinal studies emphasizing stakeholder collaboration and low-burden protocols to optimize efficiency [14]. Similar to community-engaged methods (e.g., social media, referrals) combined with traditional outreach [15]; our approach prioritized healthcare facility and community partnerships to enhance engagement, with a smooth recruitment.

The level of retention in our study was higher than reported in other similar studies examining longitudinal participant retention in SARS-CoV-2 antibody seroprevalence research across both HICs and LMICs. For instance, a study from Mekelle, Ethiopia reported a retention rate of 85%, with participants followed for a median of 31 days (interquartile range [IQR]: 16–32) and a range of 5 to 39 days [16]. A study from Nigeria among 525 participants reported a six-month follow-up rate of 93.5%, with two rounds of sample collection at 3 and 6 months [17]. Although their overall retention appears higher than our overall 88% across nine rounds over two years, a direct comparison at equivalent follow-up intervals shows that our retention at six months was 97.4%, surpassing their short-term retention. A longitudinal study assessing serological responses to SARS-CoV-2 among HCWs in Poland reported a retention rate of 83% over one year [18]. Another cohort study in Germany focusing on hospital employees achieved a retention rate of 46% over six months [19]. A study conducted in Victoria, Australia, among 1000 community based-Victorians from September 1, 2020, to September 30, 2021, achieved a retention rate of 85% over a 12-month period [20]. Similarly, research on recruitment and retention strategies in longitudinal clinical studies with low-income population participant retention rate was 75% and 64% at 6 months and 12 months post-recruitment, respectively [14]. These highlight that our strategies were effective even in a resource-limited setting with longer duration of the study period.

Key retention strategies in our study were grounded in the day-to-day experiences of the study staff and tailored to the needs of our participants. Personalized communication played a central role; our outreach team maintained regular phone contact and sent reminders ahead of each visit, fostering a strong sense of trust and connection. Flexibility was a critical element of the study’s success. Study staff accommodated participants’ work schedules by conducting visits during off-hours, weekends, and, when necessary, arranging home visits only after prior communication and confirmation of participants’ willingness, particularly for those with mobility challenges or scheduling conflicts. This responsiveness was especially valued by HCWs with demanding shifts and community members with caregiving responsibilities. To further support participation, the study implemented an extended visit window of ± 6 weeks, allowing individuals ample time to reschedule without missing their appointments. This approach significantly enhanced retention by minimizing dropouts due to conflicting responsibilities.

Overall, staff demonstrated remarkable adaptability and commitment, often going beyond routine duties to ensure participant convenience and comfort. These practical, human-centered approaches align with evidence from a systematic review that identified 95 retention strategies, categorized into barrier-reduction, community-building, follow-up/reminder, and tracing strategies [6]. Studies frequently employed similar measures, such as regular telephone or SMS reminders, transportation support or reimbursements, home visits, and incentives, and systematic evidence shows these strategies can increase follow-up rates and reduce attrition in cohort studies [5, 6]. Similarly, our experience reflects findings from a systematic review of longitudinal studies, which identified regular reminders, clear reinforcement of study benefits, and flexible scheduling as some of the most effective retention practices [21].

Despite the implementation of robust retention strategies, several challenges were encountered during both the recruitment and follow-up phases, many of which were shaped by cultural and contextual factors. During recruitment, hesitancy was observed among some individuals due to concerns or lack of clarity about the study’s purpose. In some cases, cultural norms required participants to consult with family members, particularly spouses or elders, before providing consent, which led to delays or refusals. A similar pattern has been reported in cohort and community-based studies in Africa and other low-resource settings, where participants often delayed or declined enrollment because they needed time to consult family or community gatekeepers before consenting [22, 23]. These challenges highlight the importance of culturally sensitive recruitment strategies, strong community engagement, and clear communication from the outset.

During follow-up visits, familial responsibilities such as caregiving and household duties often limited participants’ availability. Religious commitments, particularly during major observances, occasionally conflicted with scheduled appointments. Additionally, health issues such as anemia or chronic illnesses affected some participants’ ability to consistently attend follow-up visits. In some instances, participants discontinued their involvement without providing specific reasons. A systematic review of longitudinal cohort studies reported that participant burden, including competing family and work responsibilities and physical health challenges, is associated with lower retention and increased loss to follow-up [6]. These findings reflect the complex and multifactorial nature of attrition in longitudinal studies conducted in culturally diverse and resource-limited settings.

To address these challenges, the study implemented several context-appropriate strategies. These included personalized and respectful communication, flexible scheduling, use of contact locators with regular updates, and voluntary home visits with full participant consent for those unable to attend follow-up visits in person. Staff members’ experience, cultural awareness, and a friendly, accommodating approach were instrumental in fostering trust and sustained engagement. Participant compensation, approved by the IRB and carefully calibrated to local economic standards, also contributed to continued participation with full transparency during the consent process. Similarly, other research has found that flexible scheduling, maintaining up-to-date contact information, personalized follow-up, and incentives or compensation that address participant needs can improve retention in longitudinal research. In a systematic review of ethnic minority longitudinal studies, financial incentives, flexible scheduling, extensive contact information, and trust-building communication were key facilitators of retention [24]. In another study of longitudinal clinical research with high retention rates, tailored contact and scheduling strategies, emphasizing the benefits of participation, and persistent, culturally sensitive staff engagement were commonly reported components of successful retention [21]. These strategies proved effective, as reflected in the high retention rate achieved in the study.

Consistently, other studies reported similar strategies that we used for addressing retention, such as promoting positive relationships with participants [25], conveyed respect for participants [26], updating contact information at every data collection point [27], incentives were also a useful retention strategy [28, 29]. Incorporating these lessons and enhancing initial recruitment messaging and cultural sensitivity may further strengthen future cohort studies in similar settings.

### Strengths and limitations

The study benefited from a well-structured longitudinal design with repeated follow-up visits, allowing detailed tracking of SARS-CoV-2 antibody responses over time. Thoughtful recruitment planning, personalized communication, and flexible scheduling supported high participant retention and engagement. Including both HCWs and community members provided perspectives across diverse population groups, while the participant-friendly consenting process ensured voluntariness and trust. The use of multiple communication channels and home visits further facilitated consistent follow-up in a resource-limited setting.

The study also has limitations. Conducting it at a single center may limit the generalizability of the findings. The convenience sampling approach, relying on accessible participants, could introduce selection bias. Additionally, dependence on phone and in-person communication in areas with connectivity challenges may have affected participation or timely follow-up. Despite these limitations, the study offers useful insights into retention strategies and longitudinal antibody dynamics in this context.

## Conclusion

This longitudinal cohort study on SARS-CoV-2 antibody responses among HCWs and community members in Ethiopia successfully maintained high participant retention over the follow-up period. The study demonstrates that despite challenges such as mobility issues, health-related concerns, and changing participant priorities, careful recruitment strategies, personalized communication, flexible scheduling, and the establishment of a supportive rapport with participants are crucial for achieving sustained engagement in longitudinal studies, particularly in resource-limited settings. The insights gained not only enhance understanding of SARS-CoV-2 antibody responses but also provide valuable lessons for maintaining participant engagement in similar public health research. Future studies should continue to prioritize participant well-being, transparency, and adaptability to maximize retention and ensure high-quality data collection.

## Supplementary Information

Additional file 1. Sequence of events for SARS-CoV-2 longitudinal cohort study in Ethiopia (November 2022 to December 2024).

Additional file 2. Health care worker participants recruiting format.

Additional file 3. Community participants recruiting format.

Additional file 4. Invitation flyers used for the SARS-CoV-2 parent study. Note: For ethical reasons, the phone numbers of research staff are currently blocked and were used solely for study purposes: +2519****** (Outreach Coordinator), + 2519******** (Study Coordinator), + 2519******** (Study Nurse), and + 2519******** (Principal Investigator).

## Data Availability

All relevant data are within the manuscript.
