# Supplementary figures and images for: Participant recruitment and retention in a longitudinal study: experience from SARS-CoV-2 cohort in Ethiopia
Source: BMC Med Res Methodol. 2026 Mar 14;26:102. doi: 10.1186/s12874-026-02823-2 (PMC13137490; doi:10.1186/s12874-026-02823-2)

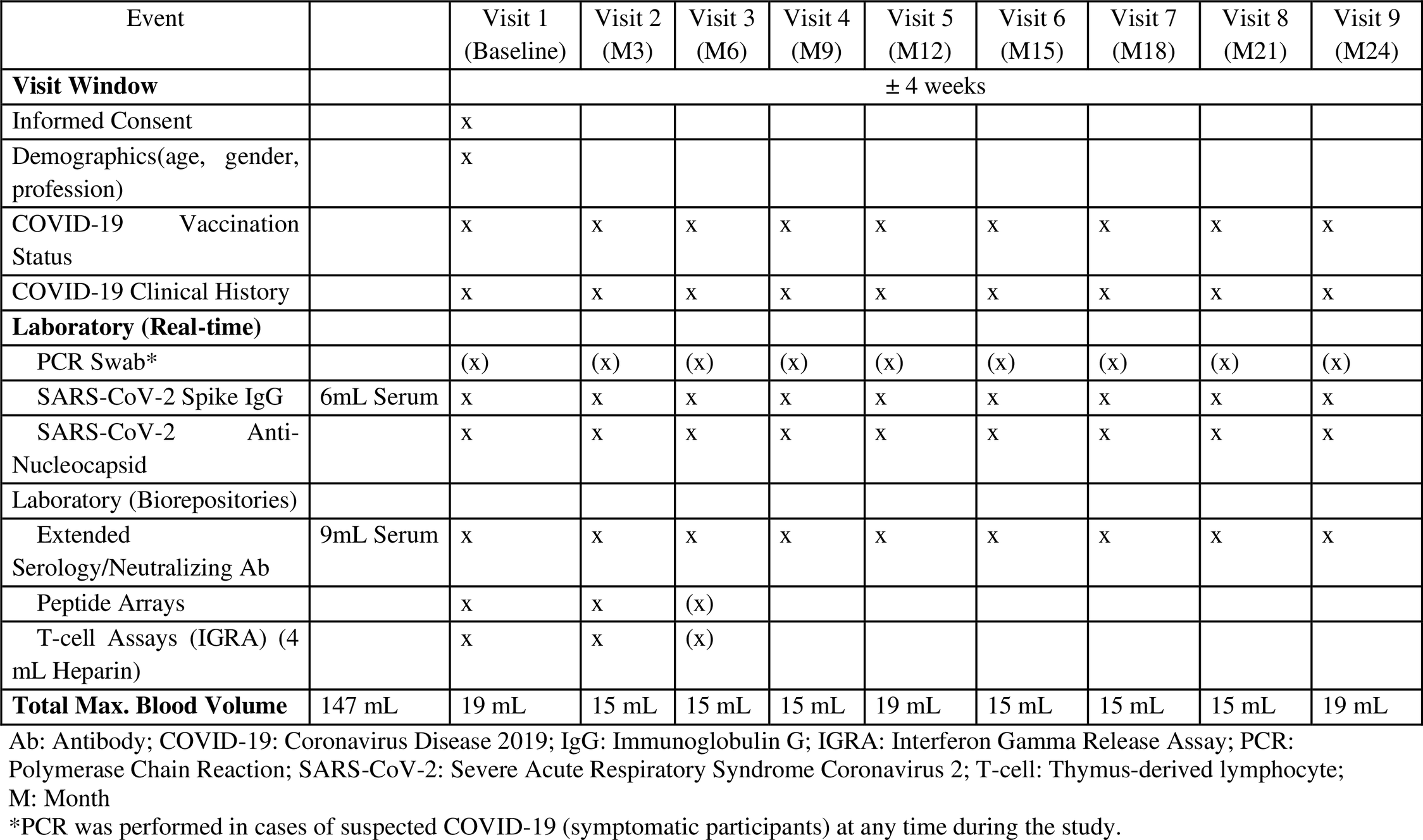

Supplement: Supplementary file 1 — Additional file 1. Sequence of events for SARS-CoV-2 longitudinal cohort study in Ethiopia (November 2022 to December 2024). [file 12874_2026_2823_MOESM1_ESM.tif]

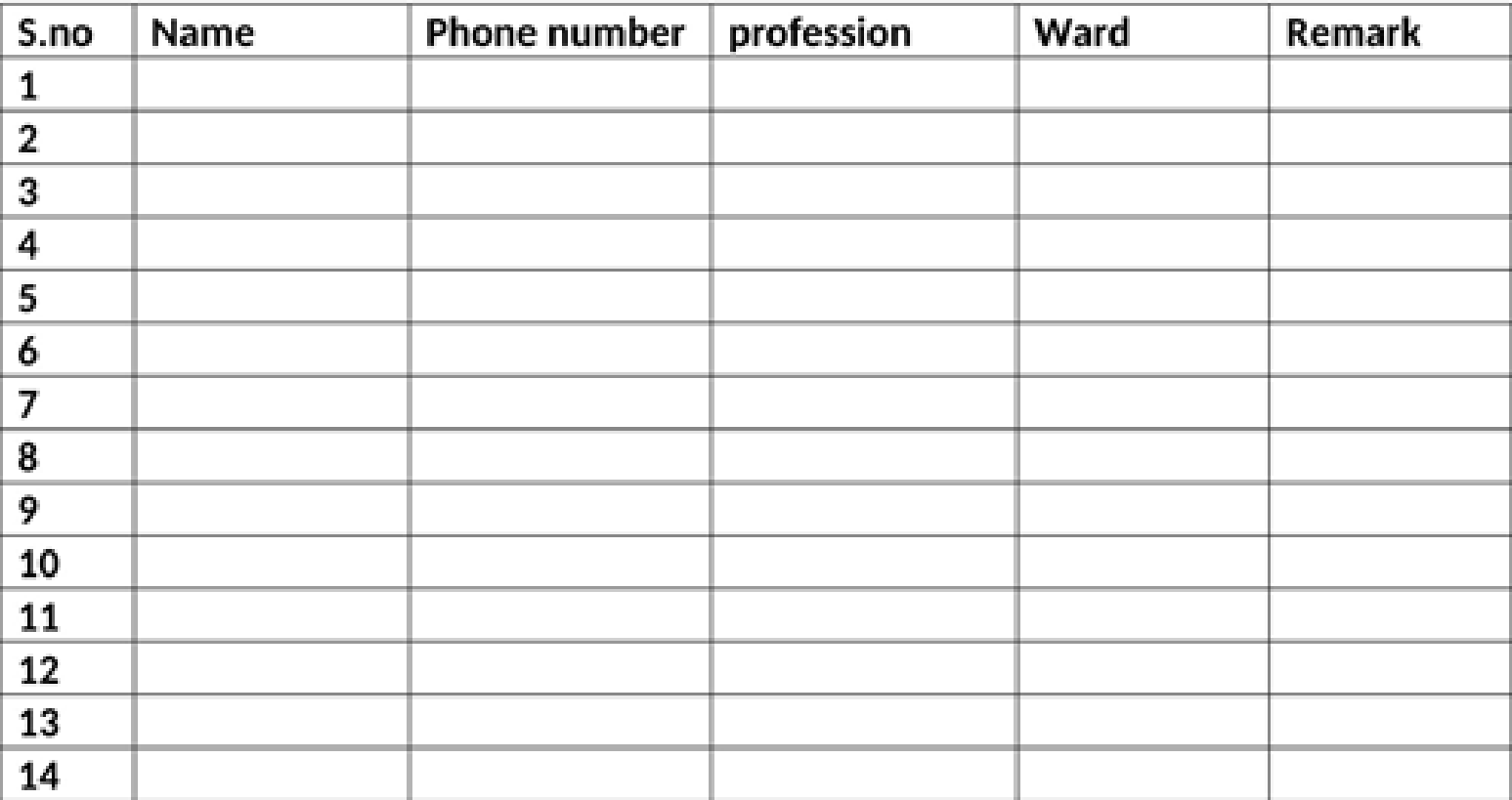

Supplement: Supplementary file 2 — Additional file 2. Health care worker participants recruiting format. [file 12874_2026_2823_MOESM2_ESM.tif]

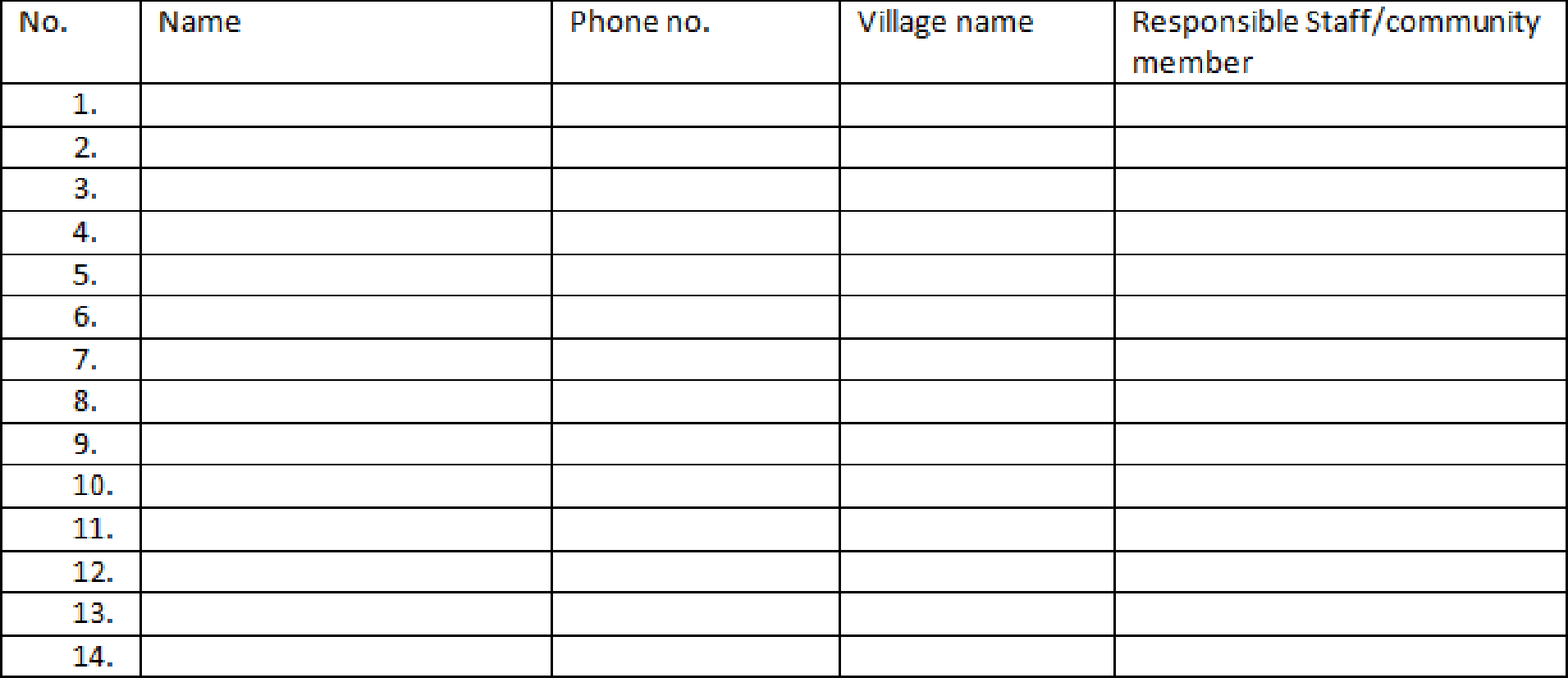

Supplement: Supplementary file 3 — Additional file 3. Community participants recruiting format. [file 12874_2026_2823_MOESM3_ESM.tif]

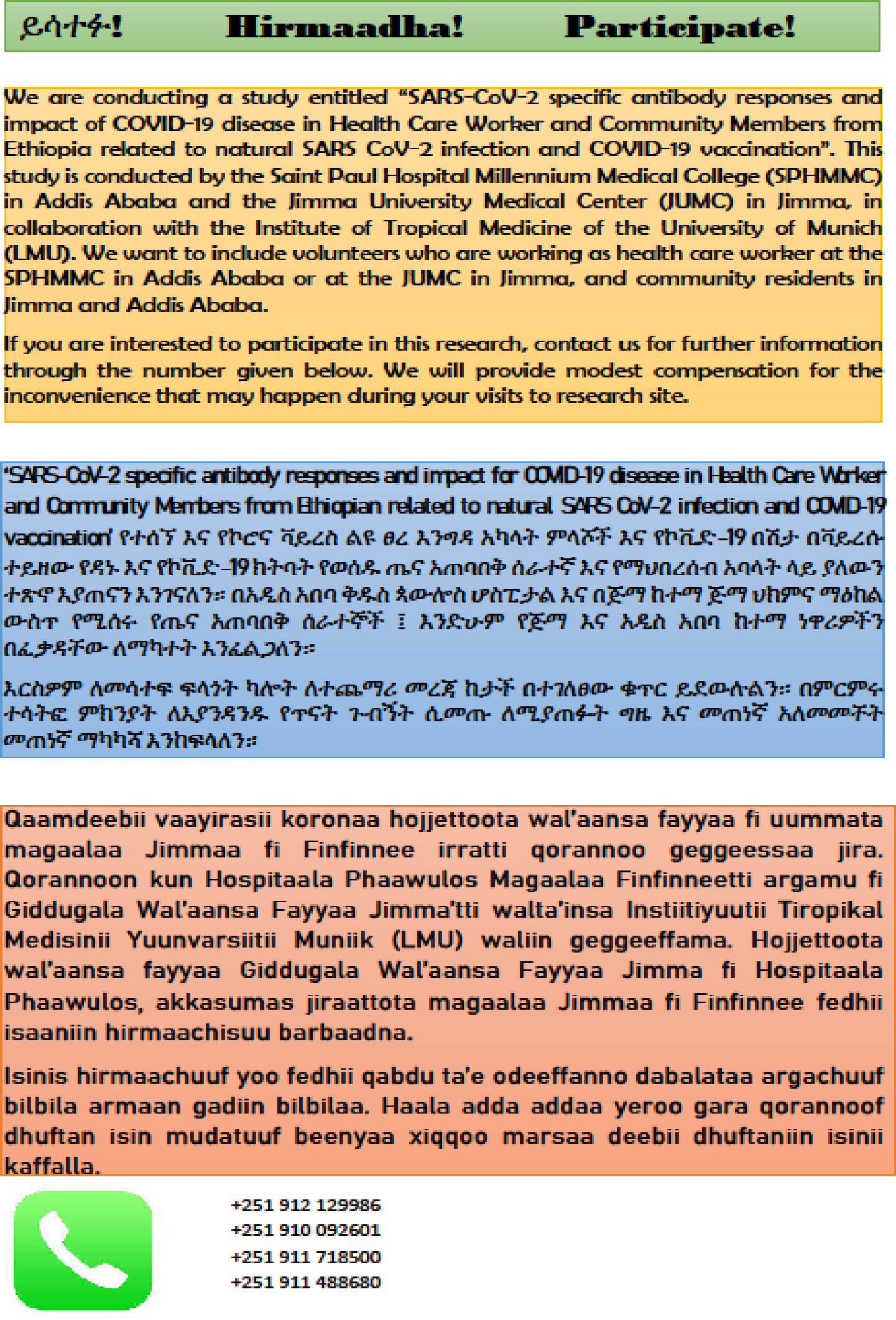

Supplement: Supplementary file 4 — Additional file 4. Invitation flyers used for the SARS-CoV-2 parent study. Note: For ethical reasons, the phone numbers of research staff are currently blocked and were used solely for study purposes: +2519****** (Outreach Coordinator), + 2519******** (Study Coordinator), + 2519******** (Study Nurse), and + 2519******** (Principal Investigator). [file 12874_2026_2823_MOESM4_ESM.tif]
